# JSONWP: a static website generator for protein bioinformatics research

**DOI:** 10.1093/bioadv/vbad154

**Published:** 2023-10-26

**Authors:** Mesih Kilinc, Kejue Jia, Robert L Jernigan

**Affiliations:** Bioinformatics and Computational Biology Program, Iowa State University, Ames, IA 50011, United States; Roy J. Carver Department of Biochemistry, Biophysics, and Molecular Biology, Iowa State University, Ames, IA 50011, United States; Bioinformatics and Computational Biology Program, Iowa State University, Ames, IA 50011, United States; Roy J. Carver Department of Biochemistry, Biophysics, and Molecular Biology, Iowa State University, Ames, IA 50011, United States

## Abstract

**Motivation:**

Presenting the integrated results of bioinformatics research can be challenging and requires sophisticated visualization components, which can be time-consuming to develop. This article presents a new way to effectively communicate research findings.

**Results:**

We have developed a static web page generator, JSONWP, which is specifically designed for protein bioinformatics research. Utilizing React (a JavaScript library used to build interactive and dynamic user interfaces for web applications), we have integrated publicly available bioinformatics visualization components to provide standardized access to these components. JSON (or JavaScript Object Notation, is a lightweight textual data format often used to structure and exchange information between different software tools.) is used as the input source due to its ability to represent nearly all types of data using key and value pairs. This allows researchers to use their preferred programming language to create a JSON representation, which can then be converted into a website by JSONWP. No server or domain is required to host the website, as only the publicly accessible JSON file is required.

**Conclusions:**

Overall, JSONWP provides a useful new tool for bioinformatics researchers to effectively communicate their findings. The open-source implementation is located at https://github.com/MesihK/react-json-wpbuilder, and the tool can be used at jsonwp.onrender.com.

## 1 Introduction

In the field of bioinformatics, the best way to effectively present research findings is a persistent challenge due to the complexity of the data and the requirement for sophisticated visualization of biological data such as structures. Although there are several solutions available, such as Plotly, they are not able to fully address the need to present bioinformatics data in a comprehensive and user-friendly manner. The use of Plotly (Plotly Technologies Inc 2015), for example, requires explicit programming and a server to run, making it too complex for most practical applications and limiting its utility for bioinformatics data presentations. Other tools such as Mobyle (Neron *et al.* 2009), Amadea (de Brevern *et al.* 2015), and Galaxy (Afgan *et al.* 2022) are systems designed to provide a flexible and usable environment for defining and running bioinformatics pipelines with simple data management features that focus more on execution, instead of the presentation of results, and lack an easy-to-use generation of websites. Other tools such as Cytoscape (Shannon *et al.* 2003), an open-source software platform used for visualizing and analyzing complex biological networks, is a powerful tool that allows researchers to explore and make sense of large-scale biological data. Moreover, extensions such as CyToStruct (Nepomnyachiy *et al.* 2015) were developed to enhance Cytoscape. CyToStruct is a tool that serves as a bridge between the Cytoscape platform and molecular visualization software such as PyMOL (Schrödinger LLC 2010), UCSF Chimera (Pettersen *et al.* 2004), VMD (Humphrey *et al.* 1996), and Jmol (www.jmol.org), allowing users to seamlessly integrate network representations of biological data with three-dimensional structural visualizations of individual molecular entities. However, these tools are primarily used in research and are not so suitable for presenting results or the findings from a study. Moreover, they require explicit software to be installed. As a result, researchers typically publish large data in Excel supplementary tables.

To address this issue, a new approach for presenting complex bioinformatics results has been developed. This solution takes the form of a JSON-based static website generator that integrates various visualization components to more effectively communicate research findings, specific to proteins. (This could also be used for RNA studies.) This approach leverages tools such as NGL.js (Rose *et al.* 2018), Ctytoscape (Shannon *et al.* 2003), and components from the NextProt project (Zahn-Zabal *et al.* 2019) and Plotly, to provide a comprehensive and user-friendly presentation of bioinformatics data. The generator utilizes the JSON data representation format, which allows researchers to use their preferred programming language to create a JSON representation of their data. The generated website can be created immediately and does not require a server or domain to host. The only requirement is that the JSON file should be publicly accessible online, and the generator can fetch this data and turn it into a website with ease. Moreover, a user does not need to install any application and can access the website even from a smartphone.

In this article, we present this new approach to presenting complex bioinformatics results, which promises a useful solution for bioinformatics data visualization.

## 2 Methods

The JSONWP tool offers a range of benefits compared to other static website generators, particularly those based on pure markdown. Markdown is a lightweight markup language that allows you to write formatted text using plain text syntax. It’s good for creating web content that is easy to read and to write. With a specific focus on bioinformatics research, JSONWP incorporates markdown and also allows for visualization of protein sequences, multiple sequence alignments, protein structures, and any tables with features such as pagination, search functionality, as well as the ability to sort and filter. Moreover, JSONWP can display Cytoscape networks. Online documentation accessible on the main page of JSONWP presents detailed information on how to use each individual functionality. It is important to note that as a static website generator, JSONWP is best suited for supplementary information rather than primary presentation of all results.

**Figure 1.**
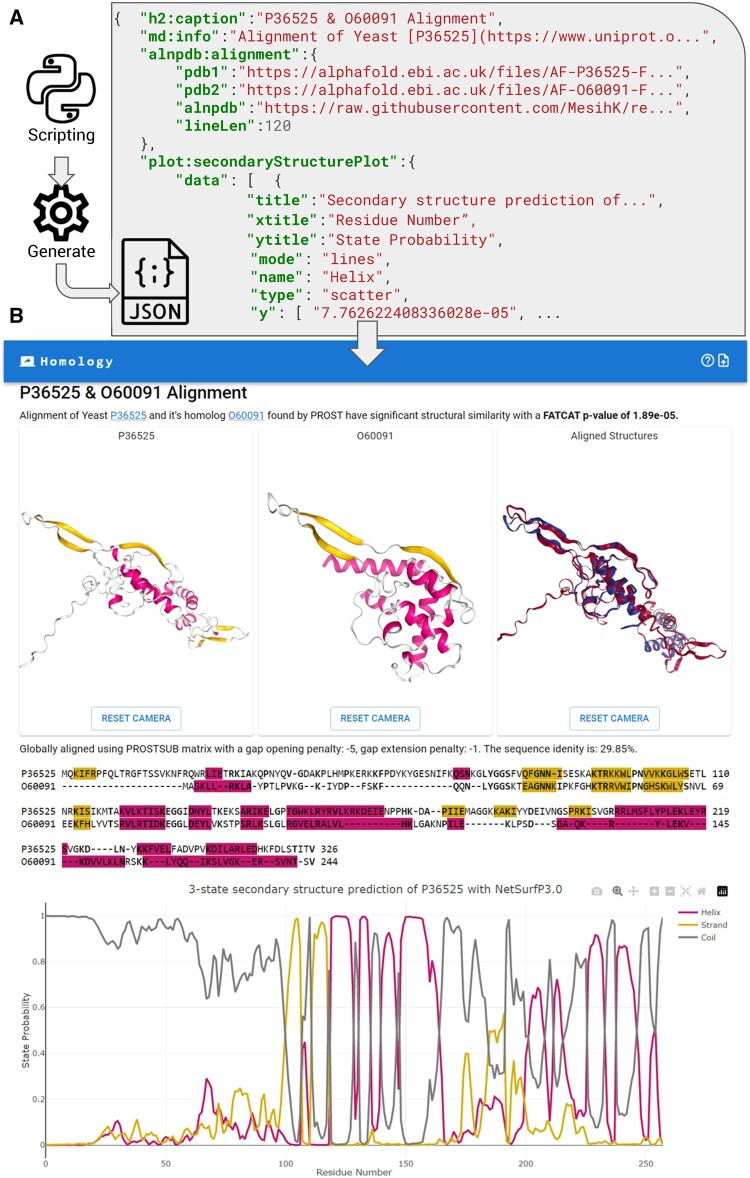
An overview of the static website generation process produced by the JSONWP tool. (A) A user can utilize any scripting language to generate a JSON representation of research results. JSON is a powerful data format, consisting of key-value pairs, which can accommodate a variety of data formats, including integers and strings, and even nested key-value pairs, as demonstrated by the example. (B) JSONWP visualization result of the JSON data. Sequence and structure alignment are shown highlighting structural features such as helices and beta sheets. Secondary structure predictions from NetSurfP3.0 are also shown as a plot.

The JSONWP tool supports the incorporation of a JSON file through either direct upload from local storage or retrieval from a publicly accessible online source, such as Github, through the use of the “? json=” argument in the URL. It is imperative to ensure that the JSON file is publicly accessible, as privately accessible files will not be functional within the tool. Upon parsing the JSON file, the JSONWP tool generates React (a web framework) components that are defined in the JSON file. The JSONWP tool possesses the ability to visualize pairs of related proteins. It accomplishes this by performing sequence alignments utilizing the ProtSub matrix (Jia and Jernigan 2021) and subsequently highlights the secondary structures within the sequence, thus making it useful for presenting the results of homology searches generated using programs such as BLAST (Altschul *et al.* 1990) or PROST (Kilinc *et al.* 2023). It is worth mentioning that JSONWP can extract sequence information from PDB data and compute the secondary assignments utilizing the TM-Align algorithm (Zhang and Skolnick 2005) on the fly with a JavaScript implementation that operates in the client browser. Figure 1 shows a visual representation of this workflow and provides an example, showcasing a practical application where a pair of homologous proteins is visualized. Utilizing the ProtSub matrix (Jia and Jernigan 2021), sequence alignment is performed automatically, and the resulting alignment is color-coded according to secondary structure. The aligned structures can be displayed by providing links to PDB files in the JSON file. In addition, the plot displaying the confidence levels of NetSurfP3.0 (Høie *et al.* 2022) tool in predicting the secondary structure of P36525 is included.

## 3 Discussion

In conclusion, the JSONWP tool provides a versatile and robust solution for the generation of static websites for protein (and RNA) bioinformatics research. With the ability to visualize sequences, structures, networks, and advanced data tables, JSONWP offers numerous advantages over other static website generators and provides a tailored approach for the unique needs of structure-sequence-based bioinformatics research.

## Data Availability

The open-source implementation can be found at https://github.com/MesihK/react-json-wpbuilder, and the tool can be used at jsonwp.onrender.com.
